# P-1673. Antibiotic Stewardship in a palliative care setting reduces antimicrobial consumption and is cost-effective

**DOI:** 10.1093/ofid/ofae631.1839

**Published:** 2025-01-29

**Authors:** Tamara K Dörr, Miriam Buschor-Bichsel, Lora Thompson, Susanne Rüfenacht, Nadia Eberhard-Kuhn, Michela Cipriani, Stefan Kuster, Philipp Kohler

**Affiliations:** Cantonal Hospital St. Gallen, St. Gallen, Sankt Gallen, Switzerland; Cantonal Hospital St. Gallen, St. Gallen, Sankt Gallen, Switzerland; Cantonal Hostpial St. Gallen, St. Gallen, Sankt Gallen, Switzerland; Lucerne Cantonal Hospital, Lucerne, Luzern, Switzerland; Cantonal Hospital St. Gallen, St. Gallen, Sankt Gallen, Switzerland; Cantonal Hospital St. Gallen, St. Gallen, Sankt Gallen, Switzerland; Cantonal Hospital St. Gallen, St. Gallen, Sankt Gallen, Switzerland; Hopital St. Gallen, St. Gallen, Zurich, Switzerland

## Abstract

**Background:**

More than 50% of patients in palliative care (PC) receive antibiotics in the final days of their life. While infection commonly occurs in this heterogenic population, clinical signs such as fever, malaise and elevation of inflammatory markers may also be attributable to the underlying disease. We evaluated the impact of a PC antimicrobial stewardship (ASP) intervention on antimicrobial consumption and cost effectiveness.

**Figure:**
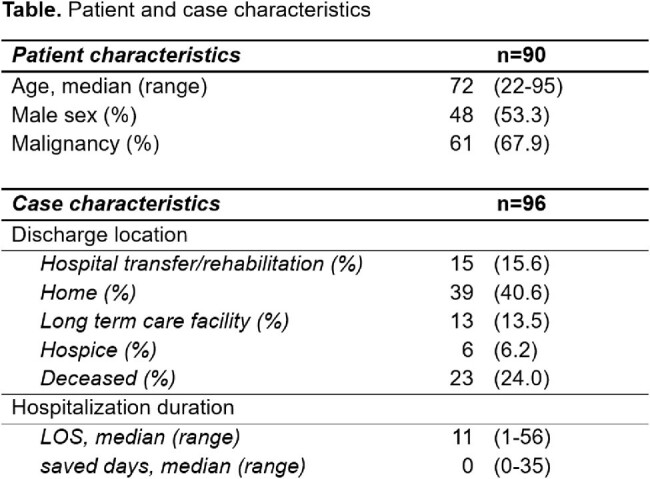

**Methods:**

Starting 08/2022, the ASP and PC team of our tertiary care hospital in Switzerland conducted an interdisciplinary stewardship intervention. During weekly visits, the ASP team provided review and feedback on cases selected by the PC physicians, recommendations made were contextualized with the patient’s superordinate goals. In added teaching sequences, general stewardship principles were explained to the PC team.

Cases were documented prospectively, gathering patient characteristics (i.e. age, sex, diagnoses), the ASP recommendation made regarding antimicrobials (i.e. none, start, continuation, oral switch, stop), its acceptance by the PC team (defined as realization within 48h) and hospitalization markers (i.e. length of stay (LOS), discharge location). Impact of the intervention on LOS and days of antimicrobials were estimated independently by two members of the ASP; disagreements were resolved by an arbiter.

Descriptive analysis was performed, associated cost savings were calculated and offset with ASP operational costs.

Proportions of recommendations made by ASP team

**Figure:**
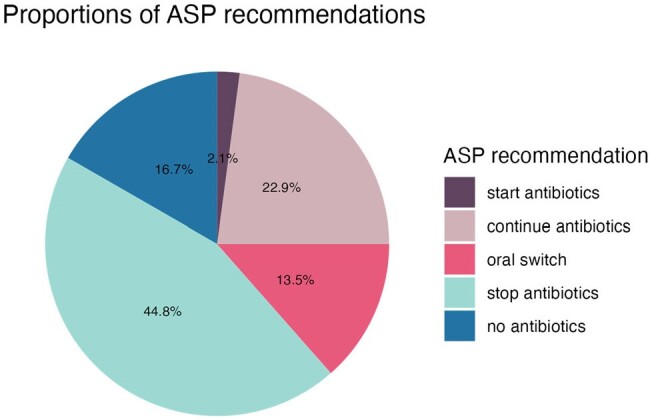

**Results:**

Between 08/2022 and 12/2023, 96 interventions were made in 90 patients (median age 72 years), malignancy was present in 61 (68%) (Table). The most frequent recommendation was to stop antimicrobials (45%), in 17% the initiation of antimicrobials was discouraged (Figure). Overall, 96% of recommendations were accepted by the PC team, leading to an overall saving of 63 hospitalization days and 177 days of parenteral antimicrobials. Calculating the net economic effect of the ASP intervention, savings of 140’314 USD were offset by 9767 USD operational cost, resulting in overall savings of 130’547 USD.

**Conclusion:**

Weekly interdisciplinary case discussions between the ASP and the PC team reduces antimicrobial consumption and hospitalization days and can be cost-effective.

**Disclosures:**

**All Authors**: No reported disclosures

